# Associations between measures of vascular structure and function and systemic circulating blood markers in humans

**DOI:** 10.14814/phy2.12982

**Published:** 2016-09-26

**Authors:** Lisa M. Cotie, Katharine D. Currie, Greg M. McGill, Austin J. Cameron, Alison S. McFadden, Stuart M. Phillips, Maureen J. MacDonald

**Affiliations:** ^1^ Department of KinesiologyMcMaster UniversityHamiltonOntarioCanada

**Keywords:** Blood markers, vascular function, vascular structure

## Abstract

Examination of relationships between systemic markers and functional measures of arterial structure and function may assist in determining alternative indices of vascular regulation and designing and evaluating interventions to improve arterial structure and function. Twenty young healthy individuals, 20 older healthy men, and 26 individuals with coronary artery disease (CAD), comprising a spectrum of vascular health, participated. Systemic markers of vascular structure and function included: pro‐collagen type I C‐peptide (PIP) – marker of collagen synthesis, C‐telopeptide of type I collagen (CTX) – marker of collagen degradation, endothelin‐1 (ET‐1) ‐ vasoconstrictor, and interleukin‐6 (IL‐6) – inflammatory marker. Functional measures of arterial structure and function included carotid artery distensibility and brachial artery flow‐mediated dilation (FMD). Moderate positive relationships were observed between carotid distensibility and CTX and PIP (*r* = 0.57, *P* < 0.0001 and *r* = 0.47, *P* < 0.0001). A negative correlation exists between ET‐1 and FMD (*r* = −0.44, *P* = 0.0004); however, no relationship was observed between IL‐6 and FMD (*P* = 0.25). Over a broad range of vascular health, relationships were observed between markers of type I collagen turnover and arterial stiffness and between a marker of vasoconstriction and endothelial function. These results indicate that regulatory links, between the indices examined, exist. Therefore, monitoring systemic markers rather than functional vascular measures, may provide sufficient information about vascular health and should be considered in the design and evaluation of vascular interventions.

## Introduction

An understanding of the relationship between blood markers linked to vascular regulation and non‐invasive tests of vascular structure and vascular function may provide useful information about the strength of the mechanistic links between these indices and the feasibility of using blood markers as surrogates for functional assessments. Commonly used functional tests for arterial structure and function include measurements of carotid artery distensibility for assessing arterial structure, and brachial artery flow‐mediated dilation (FMD) for assessing arterial function. Carotid artery distensibility, a measure of arterial stiffness, used to represent central aortic stiffness, is associated with cardiovascular disease (CVD) risk in healthy and clinical populations (Blacher et al. 1998; Barenbrock et al. 2002).

Type I collagen accounts for 60% of the vascular‐located collagen and is present in the intima, media, and adventitia of the vessel wall (Kohn et al. 2015). Relationships between systemic markers of type I collagen synthesis and degradation have been seen with indices of both central (Ishikawa et al. 2005; McNulty et al. 2006; Stakos et al. 2010) and peripheral artery stiffness (Ishikawa et al. 2005; Chatzikyriakou et al. 2008). However, there are conflicting reports on the direction and significance of this relationship in populations with differing levels of impairment in their vascular structure and function (Ishikawa et al. 2005; McNulty et al. 2006; Chatzikyriakou et al. 2008; Stakos et al. 2010; Dellegrottaglie et al. 2011).

Endothelial dysfunction may represent an early subclinical event in the development and progression of atherogenesis and is a known independent risk factor of CVD (Vita and Keaney 2002; Bonetti et al. 2003; Green et al. 2011). The dilatory capacity of the endothelium is commonly measured in the brachial artery via assessment of FMD, which is a strong predictor of cardiovascular events in asymptomatic individuals (Shechter et al. 2009) and in patients with established CVD (Kitta et al. 2009). Indeed, measurement of FMD is thought to provide independent prognostic information, which may supplement the information available from traditional risk factors (Shechter et al. 2009).

Factors regulating endothelial function are likely numerous, including, but not limited to: nitric oxide (NO), prostacyclin (PGI_2_), and endothelial‐derived hyperpolarizing factor (EDHF) (Stoner et al. 2012). Inflammation also appears to mitigate endothelial function and associations between inflammatory markers have been linked to endothelial (dys)function (Venugopal et al. 2002; Verma et al. 2004; Vita et al. 2004; Turemen et al. 2011). Nonetheless, studies have found an inverse relationship (Vita et al. 2004; Turemen et al. 2011) or no relationship (Verma et al. 2004) between inflammatory markers and endothelial function. Endothelin‐1 (ET‐1) may also be an important marker of endothelial function with pro‐inflammatory factors causing an increase in the synthesis of ET‐1 (Vila and Salaices 2005). In turn, ET‐1 reduces NO bioavailability by decreasing its production or increasing its degradation (Iglarz and Clozel 2007). Previous work has suggested that endothelial dysfunction is associated with a reduction in NO, but it is unclear whether an increased systemic ET‐1 is associated with this process (Vila and Salaices 2005).

To better understand the mechanisms associated with changes in vascular structure/function and to facilitate the evaluation of arterial structure and function, it is important to examine relationships between functional assessments of arterial structure/function and systemic blood markers. Thus, the purpose of this study was twofold. The first purpose was to determine the relationship between vascular stiffness, as measured by carotid artery distensibility and serum markers of type I collagen synthesis and degradation across a spectrum of arterial health. The second purpose was to determine the relationship between endothelial function and circulating markers of inflammation (IL‐6) and vasoconstriction (ET‐1) over a range of arterial health. We hypothesized that healthier, more elastic arteries would be positively associated with altered collagen turnover and that there would be an inverse relationship between endothelial function and markers of both inflammation and vasoconstriction.

## Methods

### Ethical approval

Informed consent was obtained in writing from all participants. This study conformed to the standards set by the Declaration of Helsinki, and the procedures were approved by the Hamilton Health Sciences ethics committee.

### Participants

Vascular and blood measures were obtained from 20 young health individuals (three women), 20 healthy older men, and 26 individuals with CAD (two women) for a total of 66 individuals representing a spectrum of vascular health. The young healthy individuals were recruited from the McMaster University undergraduate community. The individuals with CAD were recruited from the cardiac rehabilitation program at the Hamilton Health Sciences General campus. The healthy older men were recruited from the community using newspaper advertisements in the Hamilton Spectator. Each population was chosen to represent parts of the cardiovascular health spectrum. Participant characteristics are summarized in Table 1.

**Table 1:** Subgroup characteristics and mean differences

	Young *N* = 20	CAD *N* = 26	Old *N* = 20	Mean differences
Age (years)	22.4 ± 0.6	64.8 ± 1.7	69.2 ± 0.8	*P* < 0.05^a^, ^b^, ^c^
Sex	18 males	24 males	20 males	n/a
BMI (kg/m^2^)	24.1 ± 0.6	28.2 ± 0.8	27.2 ± 0.6	*P* < 0.01^a^, ^b^
Resting MAP (mmHg)	77 ± 5	86 ± 2	77 ± 3	No differences
Resting HR (bpm)	58 ± 2	59 ± 2	59 ± 2	No differences
Carotid artery distensibility	0.0065 ± 0.0005	0.0028 ± 0.0002	0.0021 ± 0.0002	*P* < 0.001^a^, ^b^
PIP (ng/mL)	1550 ± 127	919 ± 66	1088 ± 71	*P* < 0.01^a^, ^b^
CTX (ng/mL)	0.92 ± 0.1	0.39 ± 0.04	0.51 ± 0.06	*P* < 0.001^a^, ^b^
Relative FMD	6.6 ± 0.6	4.7 ± 0.5	5.1 ± 0.6	No differences
IL‐6 (pg/mL)	1.00 ± 0.14	2.31 ± 0.44	2.69 ± 0.48	*P* < 0.05^a^
ET‐1 (pg/mL)	0.74 ± 0.14	0.81 ± 0.12	0.85 ± 0.08	No differences

a Significantly different Young to Old.

b Significantly different Young to CAD.

c Significantly different Old to CAD.

### Cardiovascular measurements

#### Heart rate and blood pressure

Testing sessions began with 10 min of supine rest to ensure representative resting measurements prior to the commencement of vascular assessment. Continuous measurements of heart rate (HR) via single lead electrocardiogram (ECG) (ML123, AD Instruments Inc. Colorado Springs, CO) and blood pressure (BP) measurements for mean arterial pressure (MAP) via an automated applanation tonometer with and without oscillometric cuff calibration (Nexfin monitor, Model 1, BMEYE B.V., Amsterdam, the Netherlands or Finometer Midi, Model 2, Finapres Medical Systems, Amsterdam, the Netherlands) were made.

### Arterial structure and function measures

#### Carotid artery distensibility

Central artery stiffness was estimated with direct measurements of carotid artery distensibility using a combination of high‐resolution, two‐dimensional, brightness mode ultrasound images (Vivid Q; GE Medical Systems, Horten, Norway) and applanation tonometry (model SPT‐301; Millar Instruments, Houston, TX). Common carotid artery images were collected using a 12 MHz ultrasound probe and were collected at 23.2 frames per sec. Ultrasound images were stored offline in Digital Image and Communications in Medicine (DICOM) format for later analysis using semi‐automated edge tracking system (AMS, [Artery Measurement System] Image and Data Analysis: Tomas Gustavsson, gustav@alumni.chalmers.se). In each frame, carotid (minimum and maximum) lumen diameters were calculated from 100 measurement markers along the vessel wall within a chosen region of interest, for a total of 110,000 measures in the 10 heart cycles. A hand‐held tonometer was positioned over the point of greatest pulsation of the right common carotid artery and held in a fixed position for 10 consecutive heart cycles while ultrasound images of the left common carotid artery were collected simultaneously. Absolute carotid artery systolic BPs were calculated by calibrating the relative values acquired using applanation tonometry to the calibrated brachial artery BP acquired simultaneously using the Nexfin or Finometer (Kelly and Fitchett 1992; Nichols et al. 1998). Pulse pressure was calculated by subtracting the diastolic pressure from the systolic pressure. Distensibility was calculated using the following equation (O'Rourke et al. 2002):

(1) $$\text{Distensibility}=\frac{\left[\Pi {\left(\frac{d_{\max}}{2}\right)}^{2}-\Pi {\left(\frac{d_{\min}}{2}\right)}^{2}\right]}{\Pi {\left(\frac{d_{\min}}{2}\right)}^{2}\times \text{PP}}$$

where *d*_max_ is the maximum diameter, *d*_min_ is the minimum diameter, and PP is carotid pulse pressure.

In a small subgroup analysis, central pulse wave velocity (PWV: carotid to femoral) was measured and compared to the carotid artery distensibility measures to demonstrate the robust nature of our measures of aortic stiffness and to the serum collagen markers to strengthen observed relationships associated with vascular structure.

#### Flow‐mediated dilation assessment

An FMD test was conducted to assess brachial artery endothelium‐dependent function. With the participant in the supine position, the right arm was supinated and abducted 80–90° so that an optimal image of the brachial artery could be obtained in a comfortable position. An inflatable cuff was placed on the forearm, approximately 5 cm below the medial epicondyle (Donald et al. 2006) and remained deflated while baseline data were collected. B‐mode ultrasound images of the brachial artery were collected through two‐dimensional grayscale ultrasound imaging using a 12 MHz linear array probe (Vivid Q; GE Medical Systems) at a frame rate of 7.7 frames per sec. A baseline longitudinal image of the brachial artery (30 sec) was acquired. Simultaneous image and pulse wave Doppler was collected at all time points. The forward and reverse audio signals from the pulse wave Doppler spectrum were processed by an external spectral analysis system (Neurovision 500M, Multigon Ind; Yonkers, NY) and an intensity‐weighted calculated mean signal was acquired (Power lab model ML 795).

To create the flow stimulus, the forearm cuff was instantaneously inflated using a rapid cuff inflator (model E20 and AG101; Hokanson, Bellevue, WA) to a standardized, suprasystolic pressure of 200 mmHg (>50 mmHg above SBP) to ensure arterial inflow occlusion and ischemia of downstream vessels and tissue (Corretti et al. 2002). The cuff was instantaneously deflated after 5 min of occlusion. Similar to baseline measures, simultaneous B‐mode imaging and mean blood velocity signals were obtained for 3 min following cuff release. A semi‐automated edge detection software program (AMS, Image and Data Analysis, Tomas Gustavsson, gustav@alumni.chalmers.se) was used to detect the vessel diameters within a specific region of interest at the end‐diastolic frames of each heart cycle collected as described above. Five‐diameter rolling averages were used to calculate the peak dilation of the vessel in the 180 sec post cuff release. From this data, the absolute FMD (mm) and relative FMD (%FMD) were calculated as follows (Corretti et al. 2002):

(2) $$\begin{array}{cc} \text{Absolute FMD}= & \;\text{Peak Diameter}\text{(mm)}-\\ & \text{Baseline Diameter}\text{(mm)} \end{array}$$

(3) $$\text{Relative FMD}=\left(\frac{\text{Absolute FMD}}{\text{Baseline Diameter}}\right)\times 100\%$$

All analog signals were converted to digital by fast Fourier transform and sampled simultaneously at a sampling rate of 200 Hz using a commercially available data acquisition system (Power lab Model ML 795, AD Instruments) and software program (LabChart 7.0; AD Instruments Inc.).

### Blood analyses

Overnight fasted venous blood samples were collected at baseline from all participants for further analysis. Serum was stored at −20°C until analysis was completed. Serum samples were analyzed using ELISAs for concentrations of pro‐collagen type I (PIP) as a marker of type 1 collagen synthesis (Takara, Mountain View, CA), cross‐linked telopeptide of type I collagen (CTX) as a marker of type 1 collagen degradation (Immunodiagnostic Systems, Scottsdale, AZ), interleukin‐6 (R&D Systems Quantikine, Minneapolis, MN), and endothelin‐1 (Enzo, Life Sciences Assay Designs, Farmingdale, NY) using the corresponding immunoassay kits. Values were reanalyzed if the CVs between duplicates were greater than 10%. The final average CV between duplicates of all samples for all tests was <10%.

### Statistics

Results are presented as mean ± SE and relationships were considered significant at *P* < 0.05. Data were analyzed using Statistical Package for the Social Sciences (version 11.5 for Windows; SPSS, Chicago, IL). Descriptive statistics were used to present the individual population and pooled data means, 95% confidence intervals (95% CIs) and ranges. One‐way ANOVAs with Tukey's post hoc tests were used to assess group mean differences. Pearson correlations were used to assess relationships between carotid artery distensibility and both of the collagen markers (PIP and CTX, *n* = 66), and FMD and the inflammatory marker (IL‐6, *n* = 66) and FMD and the vasoconstrictor (ET‐1, *n* = 63).

## Results

### Populations studied

Table 1 summarizes the baseline characteristics of each population studied.

### Vascular structure

**Figure 1:**
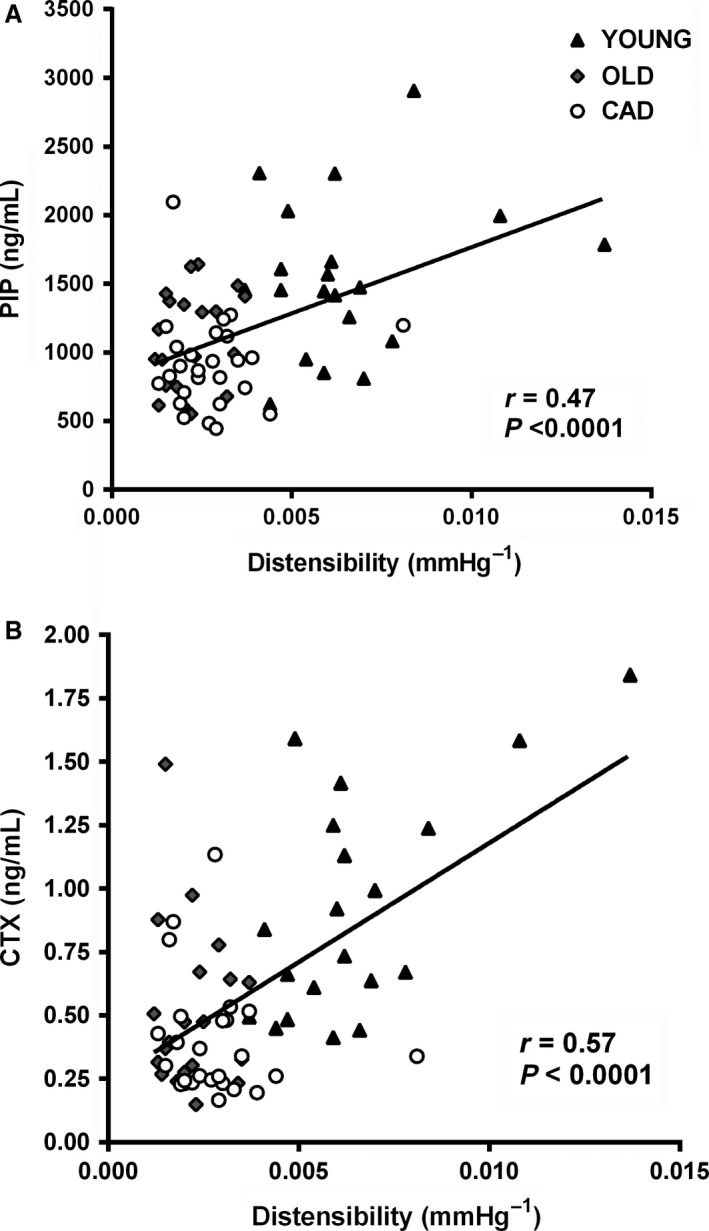
Associations between carotid artery distensibility and type I collagen markers.

There were positive relationships observed between common carotid distensibility and markers of type I collagen turnover (PIP: *r* = 0.47, *P* < 0.0001 and CTX: *r* = 0.57, *P* < 0.0001, Fig. 1). A smaller subanalysis, showed central PWV and carotid artery distensibility measures were statistically related to one another and, as expected, there was an inverse association between central PWV and carotid artery distensibility (*r* = −0.46, *P* = 0.0003, data not shown). Additionally, the subanalysis showed similar relationships between cPWV and the blood markers of collagen (PIP: *r* = −0.32, *P* = 0.04 and CTX: *r* = −0.41, *P* = 0.009).

### Vascular function

**Figure 2:**
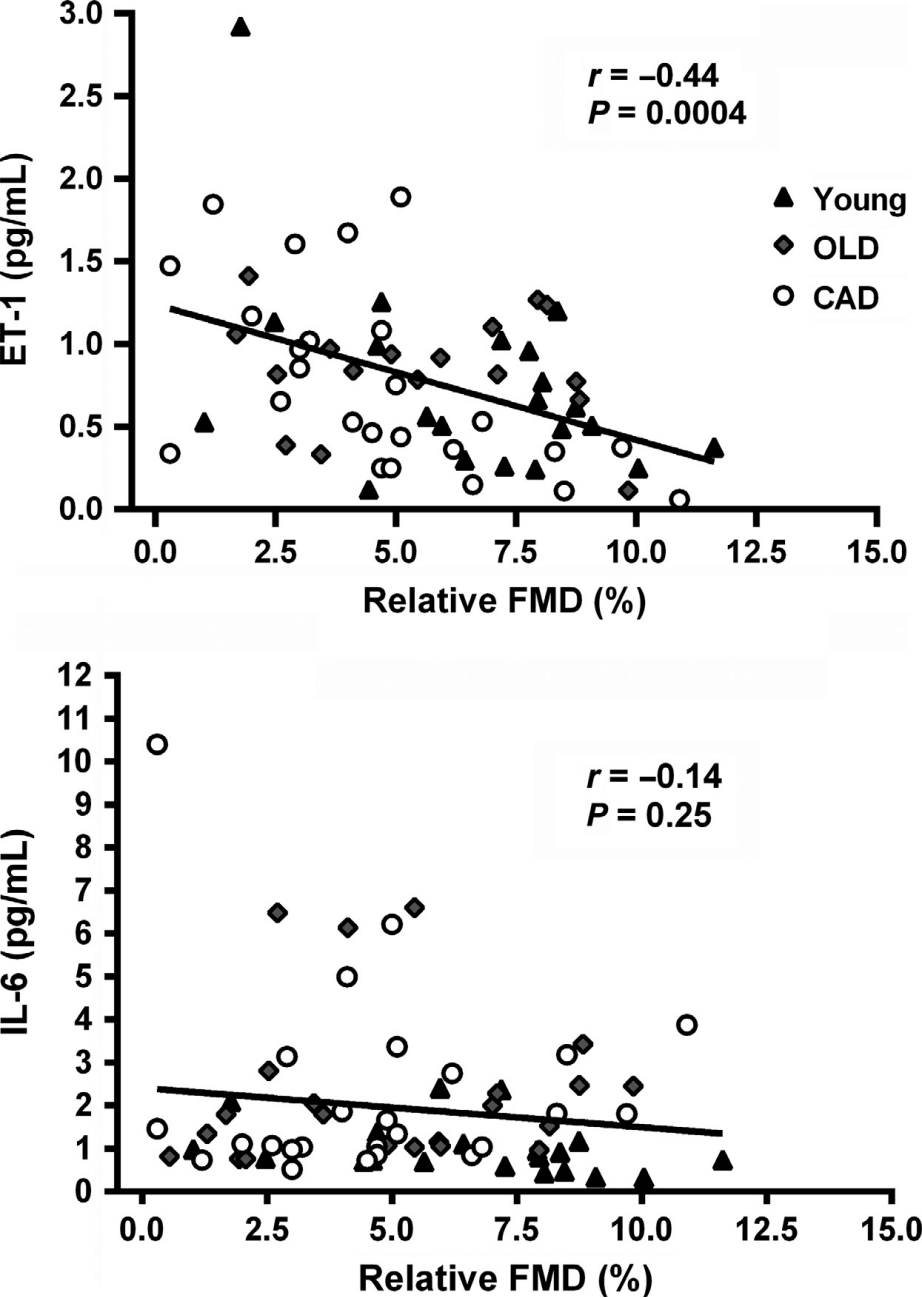
Associations between Inflammation and FMD and Vasoconstriction and FMD.

One individual in the OLD group and one individual from the CAD group had no interpretable data from the FMD test due to technical issues and a further two individuals from the OLD group had undetectable ET‐1 concentrations in their serum. Therefore, there were only 17 individuals from the OLD group and 25 individuals from the CAD group available for the FMD versus ET‐1 analysis. There was a negative correlation observed between ET‐1 and relative FMD (*r* = −0.44, *P* = 0.0004, Fig. 2). No relationships were observed between the inflammatory marker (IL‐6) and FMD (Fig. 2).

## Discussion

The most important findings in this study were that over a broad range of vascular health, relationships were observed between markers of type I collagen turnover and functional measures of arterial stiffness and between a marker of vasoconstriction and a measure of endothelial function. We chose to study relationships between vascular health and systemic markers in various populations to obtain a large range in our functional indices of arterial structure and function.

Increased carotid artery distensibility was associated with increases in both collagen breakdown (CTX) and synthesis (PIP). In agreement with some previous work, our findings suggest a link between type I collagen turnover and arterial stiffness, however, previous reports of similar relationships have been inconsistent in both the direction and magnitude of the relationship observed. Previous studies have reported both increased collagen synthesis (Ishikawa et al. 2005; Stakos et al. 2010; Dellegrottaglie et al. 2011) or decreased degradation (Chatzikyriakou et al. 2008) with increasing stiffness, and increased collagen degradation with increasing stiffness (Ishikawa et al. 2005; McNulty et al. 2006). Interestingly, our results show that markers of both collagen synthesis and degradation are augmented with improved artery elasticity suggesting that increased type I collagen turnover is associated with healthier, more elastic arteries. Changes in the composition of collagen subtypes occur in hypertensive rats with stiff arteries (Bashey et al. 1989; Chamiot Clerc et al. 1999), however, it is less clear whether this relationship exists and in what direction it exists in humans.

Previous studies in this area were conducted in relatively homogeneous populations including middle‐aged to elderly (45–90 years) clinical populations, such as stable chronic heart failure (Chatzikyriakou et al. 2008), chronic kidney disease (Dellegrottaglie et al. 2011), and medicated (Stakos et al. 2010) and non‐medicated hypertensives (Ishikawa et al. 2005; McNulty et al. 2006). Ours is the only study that has included participants with a wide range of vascular stiffness and we report that statistically significant relationships existed across this range.

Carotid artery distensibility is an index of aortic vascular structure. Carotid artery distensibility measures the inverse of stiffness in the carotid artery, which is considered to be a central elastic artery. It is important to highlight that in a small subgroup analysis (YOUNG and OLD), our measures of aortic stiffness were statistically related to one another, and as expected, there was an inverse association between central PWV and carotid artery distensibility. Additionally, central PWV was similarly related to our markers of collagen, such that stiffer arteries (higher PWV) had lower levels of PIP and CTX. These findings highlight the robust nature of our finding that markers of type I collagen turnover are associated with the functional assessments of arterial stiffness and specifically that healthier more elastic arteries are associated with greater collagen turnover.

Endothelial function is regulated by both local (Stoner et al. 2012) and systemic factors (Vita et al. 2004). We found a negative association between serum levels of the potent vasoconstrictor ET‐1 and relative FMD, suggesting that circulating markers of vasoconstriction may be indicative of the functional status of the brachial artery. Studies have shown that the functional status of the brachial artery is indicative of coronary artery functional status and overall vascular health (Anderson et al. 1995). The literature suggests that increased ET‐1 may contribute to the reduction in NO bioavailability often associated with endothelial dysfunction (Schiffrin 1995; Amiri et al. 2004; Iglarz and Clozel 2007); however, it is not currently clear whether or not ET‐1 plays a direct role in this process. Despite our inclusion of three populations, which theoretically should have a broad range of vascular health, we saw no statistically significant differences in flow‐mediated dilation between the groups (*P* = 0.07). With a broader range of vascular function, we may have observed a stronger relationship with ET‐1 and a significant relationship with IL‐6.

However, we observed no relationship between IL‐6, a marker of systemic inflammatory status, and FMD, suggesting that circulating IL‐6 may not mediate endothelial function. Contrary to our findings, Vita et al. (2004), in a cohort of 2701 participants (mean age 61 years, 53% women) from the Framingham offspring study, found a weak but significant relationship between circulating IL‐6 with FMD (Vita et al. 2004). It is possible that until there is an overt systemic inflammatory signal, such as in a disease state, no relationship exists. Nonetheless, recent research has suggested that endothelial function is largely governed by local endothelial cell‐derived factors including nitric oxide (NO), prostacyclin (PGI_2_), and endothelial‐derived hyperpolarizing factor (EDHF) (Stoner et al. 2012). Stoner et al. (2012) suggest that the relative contribution of each of these vasodilators may vary with different clinical populations. We did not measure any local markers, however, the hypothesis of primarily local factor regulation of endothelial function warrants further research.

This study increases the comprehensive understanding of factors associated with the regulation of vascular structure and function in a spectrum of populations spanning a large range of arterial structure and function. The information provided by this study may assist with focusing on direct targets, such as type I collagen turnover and vasoconstrictors to assist with lifestyle interventions designed to decrease CVD risk. The moderate relationships observed, indicate that the systemic markers assessed in this study may act as potential tools for use in comprehensive assessment of arterial structure and function.

## Conflict of Interest

None declared.
